# Determinants of the willingness to participate in biobanking among Malaysian stakeholders in the Klang Valley

**DOI:** 10.1186/s12874-018-0619-2

**Published:** 2018-12-05

**Authors:** Latifah Amin, Hasrizul Hashim, Zurina Mahadi, Khaidzir Ismail

**Affiliations:** 1 Pusat Citra Universiti, Universiti Kebangsaan Malaysia, 43600 UKM Bangi, Selangor, Malaysia; 2 Policy and Strategic Planning Division, Ministry of Energy, Science, Technology, Environment & Climate Change, 62662 Putrajaya, Malaysia; 3 Faculty of Social Science and Humanities, Universiti Kebangsaan Malaysia, 43600 UKM Bangi, Selangor, Malaysia

**Keywords:** Biobank, Willingness to participate, Determinants, Perceived benefits, Religious acceptance, Klang Valley, Malaysia

## Abstract

**Background:**

The demand in biobanking for the collection and maintenance of biological specimens and personal data from civilians to improve the prevention, diagnosis and treatment of diseases has increased notably. Despite the advancement, certain issues, specifically those related to privacy and data protection, have been critically discussed. The purposes of this study are to assess the willingness of stakeholders to participate in biobanking and to determine its predictors.

**Methods:**

A survey of 469 respondents from various stakeholder groups in the Klang Valley region of Malaysia was carried out. Based on previous research, a multi-dimensional instrument measuring willingness to participate in biobanking, and its predictors, was constructed and validated. A single step Structural Equation Modelling was performed to analyse the measurements and structural model using the International Business Machines Corporation Software Package for Social Sciences, Analysis of Moment Structures (IBM SPSS Amos) version 20 with a maximum likelihood function.

**Results:**

Malaysian stakeholders in the Klang Valley were found to be cautious of biobanks. Although they perceived the biobanks as moderately beneficial (mean score of 4.65) and were moderately willing to participate in biobanking (mean score of 4.10), they professed moderate concern about data and specimen protection issues (mean score of 4.33). Willingness to participate in biobanking was predominantly determined by four direct predictors: specific application-linked perceptions of their benefits (β = 0.35, *p* < 0.001), issues of data and specimen protection (β = − 0.31, *p* < 0.001) and religious acceptance (β = 0.15, *p* < 0.05) and trust in key players (β = 0.20, *p* < 0.001). The stakeholders’ willingness to participate in biobanking also involves the intricate relationships between the above-mentioned factors and other predictors, such as attitudes regarding technology, religiosity and engagement.

**Conclusions:**

The findings of this study reaffirmed that stakeholders’ willingness to participate in biobanking is a complex phenomenon that should be viewed from a multidimensional perspective. Stakeholder willingness to participate in biobanking is warranted when direct predictors (benefits, issues of data and specimen protection, religious acceptance, and trust in key players) as well as indirect factors are well accounted for.

**Electronic supplementary material:**

The online version of this article (10.1186/s12874-018-0619-2) contains supplementary material, which is available to authorized users.

## Background

The success of treatments for diseases caused by defective genes depends heavily upon progressive scientific research carried out by the scientific community. Over the past few decades, scientists have established strategies for identifying these genes in an effective and less expensive manner, such as by collecting as much biological and genetic information as possible to understand interactions between genotypic and phenotypic information [1]. Therefore, the need for a central organization that collects and stores numerous biological samples for use in research is crucial for meeting the demands of the researchers involved. In many countries, such an organization is called a biobank, defined as a collection of human biological samples stored and regulated for use in scientific studies by connecting samples to phenotypic and demographic data of the donors [1]. The main purpose of establishing a biobank is to link these information sources and make them accessible for various research projects aimed at enhancing the understanding of medical conditions, including their diagnosis, prevention and treatment [1].

It was reported in 2012 that more than 600 biobanks were in service in the United States, with the number of specimens stored in corresponding biobanks ranging from ten to over fifty million in the following year [1]. Concurrently, more than 400 biobanks have been operating across Europe since 2009 [2]. By 2017, the biobanking market globally was valued at $47,062 million and is predicted to reach $68,084 million by the year 2025 [3]. In Malaysia, the first biobank initiative known as “The Malaysian Cohort” was approved by the Malaysian cabinet in 2005. It involves the operation of a rich database of information and biospecimens that serves as a platform for studying the roles and interactions of genes, the environment and lifestyle patterns [4]. Through this effort, comprehensive human samples have been collected from more than 100,000 donors in Malaysia. Additionally, approximately another 39 assisted reproductive technology (ART) clinics and several tissue-specific-based biobanks have been established [5].

In most cases, biobanks usually amass and store tissue specifically for research purposes, which often includes residual samples drawn from patients over the course of clinical care. Amongst the common samples stored are serum (plasma) and solid tissue specimens; however, some biobanks also maintain peripheral blood cells or bone marrow, cord blood derivatives, pathological body fluid, cell lines, saliva, urine, stools, hair and toenails [4, 6, 7]. Prior to collecting the samples and data, participants undergo a baseline interview on their lifestyle, medical history and demographic information [4], while other physical assessments are conducted [6–8].

Two types of consent procedures are applied with regard to the inclusion of residual tissues in a biobank: opt-in and opt-out schemes. In an opt-in scheme, a person explicitly expresses his or her consent, while in an opt-out scheme, inaction is treated as a sign of consent [9]. The opt-out scheme assumes that the subject’s enrolment in a biobank (by having their samples taken), usually during a healthcare visit, denotes consent [10]. Mancini et al. [11] reported that the opt-in consent procedure has been positively perceived by most cancer patients in France. Furthermore, the opt-out scheme offers several advantages relative to the opt-in scheme, such as lower costs of operation and the scientific advantage of establishing sufficient numbers of samples to generate scientifically valid results [12]. However, the main issue surrounding the opt-out scheme concerns the possibility of using samples from the public without their knowledge and, potentially, against their will [12]. Furthermore, the opt-out scheme may create issues related to consent for researchers. Consent is viewed as a means of protecting researchers. Not obtaining explicit consent from the research participants or patients may partially limit researchers’ liability [12]. In addition, another cost-effective form of consent, broad or blanket consent, allows the researcher to use samples for research without re-contacting the donor prior to conducting research [13].

Although biobanking has been positively viewed by the public in many countries worldwide [14–22], Gaskell et al. [16] reported that the public in Central and Southern European countries maintain substantial reservations. Moreover, the public in several Islamic countries, such as Jordan [14], Saudi Arabia [15] and Qatar [18], are supportive of biobanks. Public concern regarding biobanks has been reported to centre on issues related to the protection of biological samples and personal information [23]. However, in most cases, such concerns do not hinder public support and willingness to participate in biobanking [20]. Other attributes, such as confidence in the key actors [16, 24, 25] and institutions involved in the biobanks [16, 26], have been shown to influence public attitudes towards biobanks to a greater extent. On the other hand, respondents who expressed their support for biobanks do not necessarily confirm that they would participate in such a project since participation and support are considered two different things [25]. Considering such findings, the application of biobanks to improve the prevention, diagnosis and treatment of diseases can only be successful when the public understands such a system and its implementation very well.

Perception is a process whereby a person procures an awareness or understanding of his or her environment by organizing and interpreting sensory information [27], and together with understanding and acceptance, these factors can either promote or hamper biobank advancement. Bin Abdul Aziz and colleagues [5, 28] have raised possible ethical and legal issues that may shape public trust (e.g., issues of data privacy and mishandling) in Malaysia. Hashim and colleagues [29] highlighted that although the majority of stakeholders in Malaysia view biobanking positively, they were also concerned about its possible risks, religious acceptance, and ownership and potential misuse issues. As described above, Malaysia is following global trends in establishing biobanks. To reap the benefits of such technology, Hashim et al. [29] recommended a further analysis of the causal factors of concerns related to biobanks. Once identified, these factors can help researchers and related governmental bodies devise appropriate educational and intervention programmes to enhance public awareness and to promote this promising technology.

This paper aims to assess the willingness of stakeholders in the Klang Valley region of Malaysia, to participate in biobanking and to determine its predicting factors. The stakeholders surveyed included policy makers, scientists, representatives of NGOs, religious scholars, media professionals, general public and university students. This research will be useful in understanding societal acceptance of biobanks and people’s willingness to participate in biobanking.

## Theoretical framework and hypothesis development

**Fig. 1:**
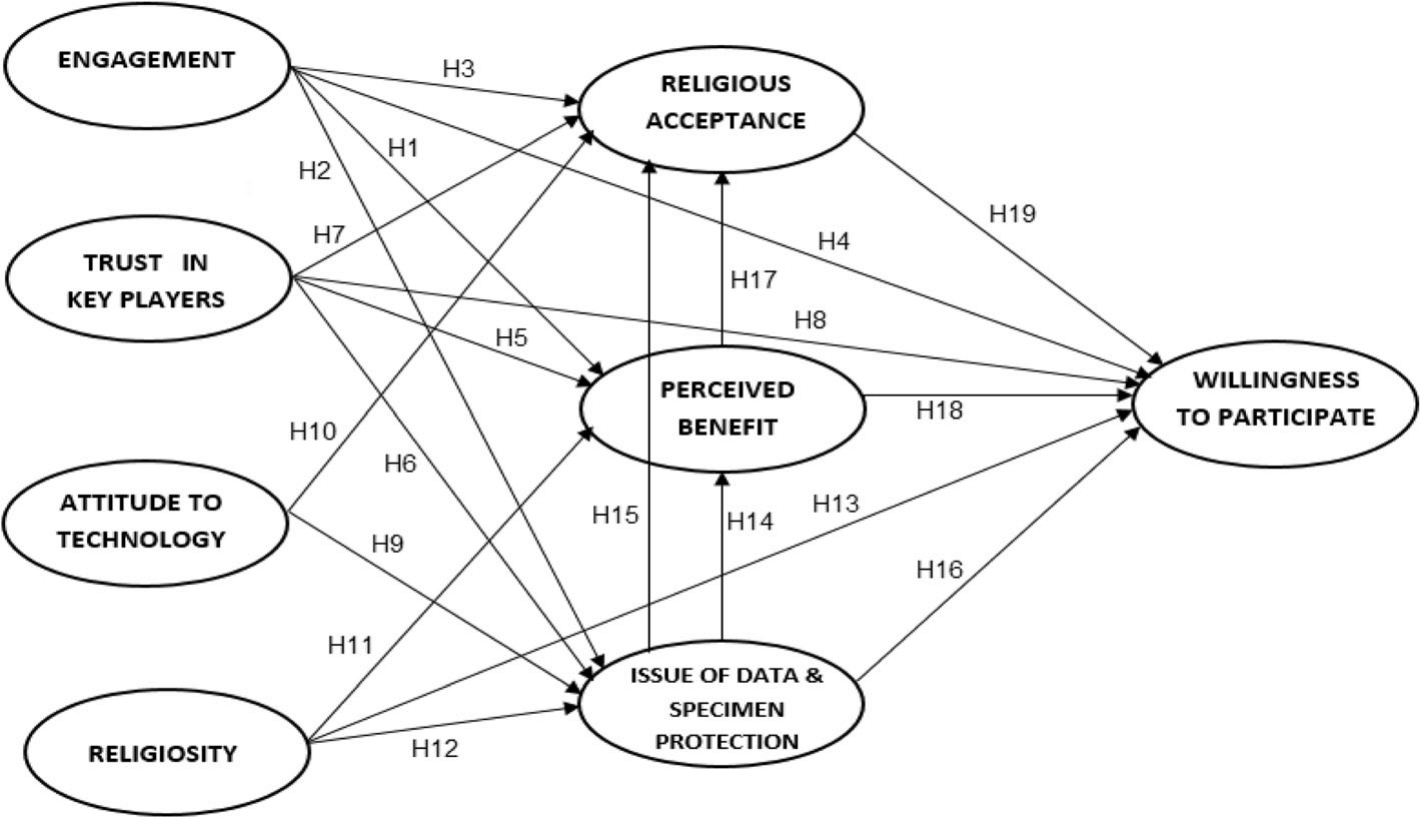
Research framework of stakeholders’ willingness to participate in biobanking

A theoretical framework of stakeholders’ willingness to participate in biobanking was designed based on the previous models of attitude to modern biotechnology applications and products [27, 30], which were the metamorphosized versions of the Fishbein’s multi-attribute attitude model [31]. Arrangement of the variables in the model is based on their presupposed influence on the subsequent variables starting with factors that have been proven to affect attitudes and behavioural intention. The magnitude of the influence of one variable on another is estimated by their regression weights. Willingness to participate in biobanking is determined by perceived benefits [32, 33], levels of religious acceptance [34, 35], and issues related to data and specimen protection [36]. General attitudinal factors such as trust in key players, engagement, religiosity and attitudes towards technology are also incorporated into the model as they have been shown to influence perceptions of benefits and risks in previous studies [32, 36–39]. A conceptual research framework of stakeholders’ willingness to participate in biobanking, labelled with the corresponding research hypotheses is presented in Fig. 1. Nineteen hypotheses were proposed based on the significant correlations between factors using bivariate Pearson correlation test, as suggested by Cheung and Chan [40] (Table 1).

**Table 1:** The matrix of correlation among the predicting factors and willingness to participate in biobanking

	Engagement	Trust in Key Players	Attitude towards Technology	Religiosity	Perceived Benefits	Issues of Data and Specimen Protection	Religious Acceptance	Willingness to Participate
Engagement	1							
Trust in Key Players	0.236**	1						
Attitude towards Technology	−0.190**	0.162**	1					
Religiosity	0.093*	0.301**	0.189**	1				
Perceived Benefits	0.316**	0.375**	−0.004	0.212**	1			
Issue of Data and Specimen Protection	−0.121**	0.212**	0.383**	0.125**	−0.116*	1		
Religious Acceptance	0.280**	0.182**	−0.189**	0.017	0.409**	−0.288**	1	
Willingness to Participate	0.152**	0.260**	−0.048	0.120**	0.414**	−0.188**	0.366**	1

*p* < 0.05*; *p* < 0.01**

### Engagement

Engagement has been demonstrated to be an important determinant of support and participation in biobanking [14], while a lack of information and understanding of genetic research has been suggested as a potential barrier to participation in biobanking [41]. This factor has also been reported as having positive association with perceived benefits and encouragement of various modern biotechnology applications and products [30, 37, 42]. In recognizing the importance of such factors to research on public perceptions, four hypotheses (H1-H4) on the association between engagement and other factors are proposed (Table 2).

**Table 2:** Research hypotheses and verification

Research hypothesis		Conclusion
H1 (Engagement ➔ Perceived benefits)	When stakeholders are more engaged with modern biotechnology, then they will perceive higher benefits associated with biobanks.	Supported
H2 (Engagement ➔ Issues of data & specimen protection)	When stakeholders are more engaged with biotechnology, then they will exhibit less concern regarding issues of data and specimen protection in biobanks.	Not Supported
H3 (Engagement ➔ Religious acceptance)	When stakeholders are more engaged with biotechnology, then they will be more accepting of biobanks from a religious perspective.	Supported
H4 (Engagement ➔ Willingness to participate)	When stakeholders are more engaged with biotechnology, then they will be more willing to participate in biobanking.	Not Supported
H5 (Trust in key players ➔ Perceived benefits)	When stakeholders have more trust in key players involved in using or regulating modern biotechnology, then they will perceive stronger benefits associated with biobanks.	Supported
H6 (Trust in key players ➔ Issues of data & specimen protection)	When stakeholders have more trust in key players involved in using or regulating modern biotechnology, then they will exhibit lower concerns regarding issues of data and specimen protection in biobanks.	Not Supported
H7 (Trust in key players ➔ Religious acceptance)	When stakeholders have more trust in key players involved in using or regulating modern biotechnology, then they will be more accepting of biobanks from a religious perspective.	Not Supported
H8 (Trust in key players ➔ Willingness to participate)	When stakeholders have more trust in key players involved in using or regulating modern biotechnology, then they will be more willing to participate in biobanking.	Supported
H9 (Attitude towards technology ➔ Issues of data & specimen protection)	When stakeholders exhibit a strong negative predisposition towards science and technology, then they will exhibit stronger concerns regarding issues of data and specimen protection in biobanks.	Supported
H10 (Attitude towards technology ➔ Religious acceptance)	When stakeholders exhibit a strong negative predisposition towards science and technology, then they will be less accepting of biobanks from a religious perspective.	Not Supported
H11 (Religiosity ➔ Perceived benefits)	When stakeholders view themselves are more religious, then they will perceive stronger benefits associated with biobanks.	Supported
H12 (Religiosity ➔ Issues of data & specimen protection)	When stakeholders view themselves as more religious, then they will exhibit stronger concerns regarding issues of data and specimen protection in biobanks.	Supported
H13 (Religiosity ➔ Willingness to participate)	When stakeholders view themselves as more religious, then they will be more willing to participate in biobanking.	Not Supported
H14 (Issues of data & specimen protection ➔ Perceived benefits)	When stakeholders have strong concerns regarding issues of data and specimen protection associated with biobanks, then they will perceive fewer benefits associated with biobanks.	Not Supported
H15 (Issues of data & specimen protection ➔ Religious acceptance)	When stakeholders have stronger concerns regarding issues of data and specimen protection associated with biobanks, then they will be less accepting of biobanks from a religious perspective.	Supported
H16 (Issues of data & specimen protection ➔ Willingness to participate)	When stakeholders have strong concerns about issues of data and specimen protection associated with biobanks, then they will be less willing to participate in biobanking.	Supported
H17 (Perceived benefits ➔ Religious acceptance)	When stakeholders perceive stronger benefits associated with biobanks, then they will be more accepting of biobanks from a religious perspective.	Supported
H18 (Perceived benefits ➔ Willingness to participate)	When stakeholders perceive stronger benefits associated with biobanks, then they be more willing to participate in biobanking.	Supported
H19 (Religious acceptance ➔ Willingness to participate)	When stakeholders are more accepting of biobanks from a religious perspective, then they will be more willing to participate in biobanking.	Supported

### Trust in key players

Since lay people are usually not well verse with the latest development in science and technology, they are not able to assess the benefits and risks of new technology directly and have to rely upon information supplied by experts. Past studies have shown that consumers’ confidence and trust in science and its regulators are related to their perceived benefits and risks of modern biotechnology applications [37, 43]. Trust in researchers at universities, hospitals and biobank institutions is an important determinant of intentions to participate in biobank research [16, 25]. Chen and Li [32] also reported that social trust in related institutions is a predicting factor for perceived risks and benefits. Thus, four hypotheses (H5-H8) were added to predict the association between trust in key players and other predictors (Table 2).

### Attitudes towards technology

Attitude towards technologies in general has been recommended as an important predictor for public support towards more specific applications such as modern biotechnology [38]. An earlier study of attitudes towards biotechnology applications in Malaysia reports that respondents who perceive science and technology negatively tend to harbour more general concerns, view GM products as having fewer benefits, more risky, have stronger moral concerns, lower risk acceptance and lower support [37]. Two hypotheses (H9 and H10) on predictive role of attitudes towards technology are proposed (Table 2).

### Religiosity

Stakeholders in Malaysia claim to be highly religious [36]. A previous study reports that highly religious Malaysians are more critical of issues related to GM foods, as they recognize their benefits, while at the same time identifying the associated risks [36]. Since religious beliefs are important to the assessment of public opinions regarding new technologies or issues, three related hypotheses (H11 - H13) are proposed (Table 2).

### Issues of data and specimen protection

The relationship between concerns over data privacy and specimen protection and support for biobanks has been well established [16]. The European public has expressed its distrust in the capability of data protection systems whilst at the same time believing that whatever is coded in a computer can always be decoded with a computer [8]. In contrast, people’s concerns about privacy issues do not necessarily lead to a rejection of biobanks [16, 26], as individuals expect biobanks to offer the best possible protections against data abuse and trust and believe in their benefits. Noting the importance of this variable, three related hypotheses (H14-H16) are proposed (Table 2).

### Perceived benefits

Perceived benefits and risks have been vastly cited as important predicting factors of public attitudes [27, 38, 44–48]. Public willingness to participate in biobank research is driven by potential for benefit sharing [16, 25] (e.g., cures for diseases) [4]. Amin and colleagues [43] highlighted that stakeholders in Malaysia assessed the beneficial aspects of modern biotechnology application along with acceptance from a religious perspective. Islam is the most widely practised religion in Malaysia, and a Muslim’s highest priority is to preserve *Shari’ah (*Islamic Law*)*, which prescribes permissible and prohibited things and actions in human life [49]. In essence, Islam encourages the use of science, technology and medicine for the betterment of human life and for the mitigation of hardships, provided that the application of such technologies brings benefits (*maslahah)* and minimizes harm (*mafsadah*) to society, the environment, and planet Earth [49]. In recognizing the important role of benefits, two related hypotheses (H17 and H18) are proposed (Table 2).

### Religious acceptance

According to previous studies, biobank research has been faced with less religious and cultural resistance from the public [17]. Nasrella and Clark [18] also found that Qatari nationals viewed volunteering in biobanking as a charitable act that is compatible with Islam and that helps future generations. Ahram et al. [50] reported that more than 60% of Jordanians believe that religious permission to make biospecimen donations for research purposes has had a positive influence on biobank participation. Additionally, the views of Malay-Muslims in Singapore and of Muslims in the United Kingdom (UK) towards biospecimen donation and biobanking were shown to be negatively shaped by presumed religious beliefs [51, 52]. Considering the significant role of religious acceptance in shaping intentions to participate in biobanking, hypothesis H19 is proposed (Table 2).

## Methods

### Survey data collection

Surveys were carried out face-to-face between March and December 2012 on 469 adults staying in Klang Valley, Malaysia. The location is selected due to its status as Malaysian social economy hub. Furthermore, those who reside in this area come from diverse backgrounds, thus meeting this study’s requirements. The respondents of the study were stratified based on stakeholder group, consisting of policy makers, scientists, representatives of NGOs, religious scholars, media professionals, the general public and university students. As recommended by Kelley [34], the respondents were given briefings on the basics of modern biotechnology prior to completing the survey questionnaires.

### Instrument

Based on previous research, an instrument to measure willingness to participate in biobanking, and its predictors was constructed. The final instrument considers eight variables, four general attitudinal factors consisting of engagement, trust in key players [38], attitudes towards technology [38, 53] and religiosity [34, 36, 38]. The other four specific variables include perceived benefits [54], issues of data and specimen protection [2], religious acceptance [34, 35] and willingness to participate [2, 16, 24]. The items are displayed in Additional file 1: Appendix.

Engagement (α = 0.691) was measured using the combination of three sub-variables: past and intended information seeking behaviours related to modern biotechnology, awareness and knowledge. The first sub-variable, which consists of five items reflecting intended and past behaviours to gather information [55], was assessed on a scale with 7-point ranging from 1 (strongly disagree), to 7 (strongly agree). The concept recommended by Gaskell et al. [55] was adopted in developing the measure for awareness. The items include awareness of eight latest developments in modern biotechnology and the national policy and regulation. The knowledge construct consists of ten statements citing basic concepts about biotechnology [56]nwith the replacement of the first item, “it is impossible to transfer animal genes into plants,” with “there are useful bacteria which live in our body”. For one item, the term “beer” from the original statement “yeast for brewing beer consists of living organisms” was changed to “bread” to suit the local culture, as most of the respondents are Muslims and therefore do not drink beer. These two sub-variables, awareness and knowledge, were assessed using dichotomous scales. Acknowledging the three sub-constructs were measured using differently, the items were recoded to a similar 10-point scale. Higher scores denote higher levels of stakeholder engagement.

The construct trust in key players (α = 0.832) was assessed by three items whether the scientists, producers and policymakers have done a good job for the Malaysian society. A 7-point scale ranging from 1 (strongly disagree) to 7 (strongly agree) was adopted. Higher scores denote higher levels of confidence in key players.

Attitude towards technology (α = 0.881) was measured by tfour items: two items on possible detrimental impact of science and technology on humanity; an item on the impact of industry and technology on urban life; and another item describing the impact of modern technology on nature. A 7-point scale ranging from 1 (strongly disagree) to 7 (strongly agree) was used. Higher scores denote stronger views on the negative impacts of technology.

Religiosity (α = 0.947) was measured with four items: three items on the importance of religion, praying and reading scriptures in life; and an item on the importance of religious views in decision making on controversial issues. A 7-point scale ranging from 1 (strongly disagree) to 7 (strongly agree) was adopted. Higher scores denote higher levels of religiosity.

Perceived benefit (α = 0.796) was assessed by four items: enhancement of quality of life and the usefulness of biobanks to Malaysian society; providing solutions to unresolved problems by traditional methods; and the balance of benefits over risks. A 7-point scale ranging from 1 (not very useful / strongly disagree) to 7 (very useful / strongly agree) was included. Higher scores denote stronger perceived benefits of biobanks.

The issue of data and specimen protection (α = 0.714) was measured with three items: biobanks may give rise to unknown consequences; worries about the ownership issue of biobank data and specimens; and the probability data and specimen misuse by researchers is high. A 7-point scale ranging from 1 (not worried at all / strongly disagree) to 7 (very worried / strongly agree) was incorporated. Higher scores denote higher levels of concern regarding issues of data and specimen protection.

Religious acceptance (α = 0.838) was measured with two items: biobanks can be accepted by my religion and biobanks can be accepted by my customs. Each item was measured on a 7-point scale ranging from 1 (strongly disagree) to 7 (strongly agree). A higher score denotes a stronger perception that biobanks could be accepted by one’s religion.

Willingness to participate (α = 0.810) comprised three items: How much do you support the sharing of personal information and biological materials among the biobanks in Malaysia?; I am willing to provide information about myself to a biobank; and I am willing to donate blood or tissue samples to a biobank. Each item was assessed on a 7-point scale ranging from 1 (not supportive at all / not willing at all) to 7 (very supportive / very willing). Higher scores denote greater willingness to participate in biobanking.

### Statistical analysis

In order to assess the consistency and uni-dimensionality of the construct, confirmatory factor analysis and tests for reliability were conducted using SPSS version 20. Bivariate Pearson correlation analyses were performed subsequent to structural equation modelling (SEM) analysis to determine relationships among the factors. SEM has been proven to be able to test a huge number of endogenous and exogenous variables simultaneously [56]. As suggested by Hair et al. [57], a single step SEM analysis using IBM SPSS Amos version 20 with the maximum likelihood function was performed to estimate both the measurement and structural model [57].

## Results

### Descriptive analysis

Before assessing the role of the various factors in predicting intention to adopt biobanks, a descriptive analysis was performed. In general, the mean scores for majority of the factors examined in this study were moderate except for religiosity, for which we found a higher mean score (Table 3). The higher mean score found for religiosity shows that regardless of their faith, Malaysians in the Klang Valley region are deeply religious.

**Table 3:** Mean scores for willingness to participate in biobanks and its predictors

Dimension	Mean score ± Standard deviation	Interpretation
Engagement	*4.50 ± 2.25	*Moderate
Trust in key players	4.89 ± 1.06	Moderate
Attitudes towards technology	4.47 ± 1.24	Moderate
Religiosity	6.12 ± 1.23	High
Perceived benefits	4.65 ± 1.13	Moderate
Issues of data and specimen protection	4.33 ± 0.93	Moderate
Religious acceptance	4.21 ± 1.29	Moderate
Willingness to participate	4.10 ± 1.25	Moderate

1–2.99: low, 3.00–5.00: moderate, 5.01–7.00: high; *0–3.33: low, 3.34–6.66: moderate, 6.67–10: high

### Measurement model

In order to test the adequacy of the measurement model, a confirmatory factor analysis (CFA) was conducted [58] which produced eight constructs. The correlation matrix for all the constructs after bivariate Pearson correlation analyses is shown in Table 1. The research hypotheses exhibited in Fig. 1 have been formulated using the data.

### Structural equation modelling

The causal relationships among variables can be determined using a powerful multivariate analysis tool, SEM [59–61]. SEM has the advantage over other general linear Models (GLMs) as it is capable of identifying the relationships between various latent constructs depicted by their measurements [62]. A structural equation model for this research was created based on assumptions from previous studies and the results of bivariate correlations among the constructs.

The model was specified using the model generation strategy recommended by Joreskog and Sorbom, but the nested models were only modified when they were substantively meaningful [63]. Four nested models were examined to determine the best model for measuring intention to participate in biobanks (Table 4). The initial model was generated and specified according to the conceptual framework presented in Fig. 1. It contains nineteen proposed hypotheses that were analysed to determine the relationships between the variables. In SEM it has been suggested that non-significant parameters be removed from the original model and to include additional paths suggested by the modification index for the purpose of improving the model fit as long as they are corroborated by theory [64]. In applying model 1, 6 of 19 hypotheses were discarded as they were not statistically significant at a probability level of 0.05. The changes made were then saved as model 2. In model 2, standardized residual co-variances for each pair of items (observed variables) were observed, and those displaying a value of above 2.5 were deleted [57, 65]. Item 21 and item 23 which represented issues of data and specimen protection exhibited a number of high residual co-variances with other items; therefore, they were excluded. Due to these changes, another two pathways were found to be not significant (Engagement ➔ Issues of data and specimen protection; Issues of data and specimen protection ➔ Perceived benefits). The model fit was improved dramatically and was later saved as model 3 (Table 4). Modification indices (MI) were examined to ensure the inclusion of any potential pathway in the model. Those presenting significantly high values of MI were considered and added to the model. At this stage, one MI was recommended (Religiosity ➔ Issues of data and specimen protection), leaving 12 pathways in the model. This pathway was originally removed when applying model 1 together with the other five pathways but was included again, as recommended by the MI. Correlated errors found among items of the same construct were allowed [64]; thus, three correlated errors were added (between e9 of item 9 and e10 of item 10, between e17 of item 17 and e18 of item 18, and between e19 of item 19 and e20 of item 20). The model was then named model 4 and was deemed the final version of the willingness to participate scale of the biobanking model. Table 2 summarize the results of hypothesis testing.

**Table 4:** Model comparison

Fit Index	Model 1	Model 2	Model 3	Model 4
χ^2^	823.8	839.6	660.0	600.8
*df*	325	332	281	279
χ^2^*/df*	2.535	2.529	2.349	2.153
*RMSEA* *(confidence interval)*	0.057 (0.052–0.062)	0.057 (0.052–0.062)	0.054 (0.048–0.059)	0.049 (0.044–0.055)
*GFI*	0.887	0.885	0.902	0.912
*AGFI*	0.859	0.859	0.878	0.889
*CFI*	0.927	0.925	0.941	0.950
*NFI*	0.885	0.883	0.902	0.910
*NNFI (TLI)*	0.915	0.915	0.931	0.941

Hair et al. [65] and Arbuckle and Wothke [66] proposed that a well-fitting and robust model should have a goodness-of-fit index (GFI) and a comparative fit index (CFI) value of greater than 0.90, whereas the root mean square error of approximation (RMSEA) should less than 0.05 and supported with a narrow confidence interval. Schumacker & Lomax [67] suggested that an adjusted goodness-of-fit index (AGFI) of above 0.70 denotes good model fit. Other than these four fit indexes, Costa-Font and Gil [68] used several commonly used fit indexes to assess the overall model fit, including the chi-square (χ^2^), CMIN/DF (χ^2^/df), normed fit index (NFI) and non-normed fit index (NNFI), whereas Carmines and McIver [69] suggested that a good model is denoted by a χ^2^/df value of less than 3. The measurement model for willingness to participate in biobanking was found to present a good fit with CMIN/DF = 2.153, CFI = 0.950, GFI = 0.912 and RMSEA = 0.049. Summary of fit indexes generated for each progression during the model development are presented in Table 4.

### Construct reliability and validity

In this study, the item and construct reliability, and internal consistency (Cronbach’s alpha) were determined. All constructs exhibited Cronbach’s alpha coefficients exceeding a value of 0.59, which are considered good (Table 5). Additionally, all items in each dimension showed corrected item-total correlations coefficients greater than 0.4, which are also categorized as good (Table 5). Composite reliabilities and the average variance extracted (AVE) values in Table 5 represented the validity of the constructs. Considering the composite reliabilities of all constructs are above 0.6, and the variances extracted (AVE) are above 0.45, it can be deducted that the constructs possessed strong convergent validity [57].

**Table 5:** Measurement scales, reliability and validity of the constructs

Factor and Item		Corrected item-total correlation	Alpha (α)	Standardized factor loading	Composite reliability	Average variance extracted (AVE)
Engagement			0.691		0.713	0.456
1.	Past and intended behaviour	0.468		0.707		
2.	Awareness	0.568		0.844		
3.	Knowledge	0.527		0.734		
Trust in Key Players			0.832		0.835	0.629
4.	Scientists have done a good job for society	0.642		0.719		
5.	Producers have done a good job for society	0.737		0.834		
6.	Policy makers have done a good job for society	0.698		0.866		
Attitudes towards Technology			0.881		0.876	0.639
7.	Leading to humanity’s extermination	0.726		0.823		
8.	Impacts on urban life	0.794		0.876		
9.	Detrimental to humanity	0.762		0.860		
10.	Upsetting the balance of nature	0.688		0.811		
Religiosity			0.947		0.949	0.823
11.	Importance of religion	0.904		0.930		
12.	Decisions made based on religious views	0.811		0.868		
13.	Importance of praying	0.911		0.942		
14.	Importance of reading scriptures	0.869		0.914		
Religious Acceptance			0.838		0.840	0.723
15.	Accepted by religion	0.722		0.852		
16.	Accepted as part of customs	0.722		0.841		
Perceived Benefit			0.796		0.774	0.464
17.	Enhance the quality of life	0.587		0.730		
18.	Useful to society	0.663		0.785		
19.	Solve problems that cannot currently be solved with traditional methods	0.528		0.696		
20.	Benefits exceed risks	0.659		0.774		
Issues of Data and Specimen Protection			0.594		0.616	0.356
22.	May give rise to unknown consequences	0.402		0.493		
24.	Worries about ownership issues related to biobank data and specimens	0.451		0.825		
25.	Probability of data and specimen misuse	0.464		0.788		
Willingness to Participate in Biobanking			0.810		0.834	0.641
26.	Support the sharing of personal information and biological materials	0.482		0.767		
27.	Willing to provide personal information	0.780		0.841		
28.	Willing to give blood or tissue samples	0.740		0.805		

### Relationships between the constructs

**Fig. 2:**
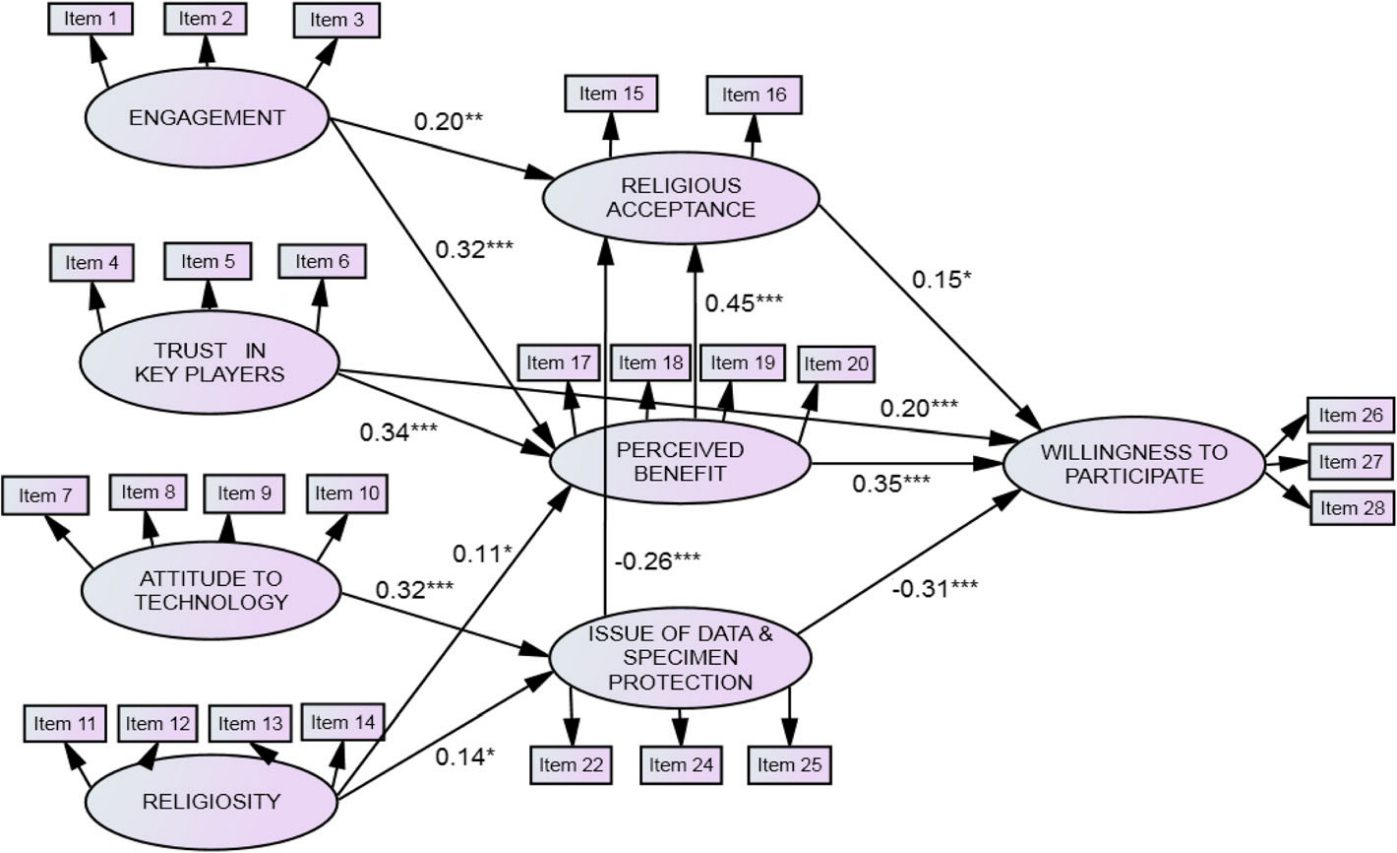
Structural equation model of factors influencing public willingness to participate in biobanking showing interrelationships among variables. Standardized estimates are presented. * *p* < 0.05, ** *p* < 0.01, *** *p* < 0.001

The final structural model for stakeholders’ willingness to participate in biobanking is presented in Fig. 2. The most important direct predictor of stakeholders’ willingness to participate in biobanking is perceived benefits (β = 0.35, *p* < 0.001). This result shows that Malaysian stakeholders in the Klang Valley predominantly assessed the beneficial aspects of biobanks when making decision whether to support the application. Issues related to data and specimen protection emerge as the next important predictor of stakeholders’ willingness to participate in biobanking (β = − 0.31, *p* < 0.001) (Fig. 2). The factor consists of several items reflecting certain risks regarding their application, including issues of data and biological sample ownership, the probability of such data and samples being misused by the authorities, and concerns regarding unknown consequences that may arise from their application.

Trust in key players came out third as a direct predictor of stakeholders’ support for and participation in biobanking (β = 0.20, *p* < 0.001) (Fig. 2). This result shows that when Malaysian stakeholders in the Klang Valley have more trust in key players involved in modern biotechnology product development or its regulation, then they will be more willing to participate in biobanking. In addition to perceived benefits, issues of data and specimen protection, trust in key players, and religious acceptance also have a positive influence on willingness to participate in biobanking (β = 0.15, *p* < 0.05) (Fig. 2). This finding suggests that Malaysian stakeholders in the Klang Valley also considered their religious judgements together with perceived benefits, issues of data and specimen protection, and trusts in key players, when deciding whether or not to support biobank application. It is important to note that religious acceptance also acts as an intermediary of the relationship between perceived benefits and willingness to participate in biobanking (β = 0.45, *p* < 0.001) (Fig. 2).

Other than direct relationships, stakeholders’ willingness in biobanking also involves the interplay of other factors. In this study, issues of data and specimen protection were found to be negatively associated with religious acceptance (β = − 0.26, *p* < 0.001), suggesting that stakeholders who view biobanks as risky tended to perceive their application as less acceptable according to their religious and cultural views (Fig. 2). Engagement is also indirectly and positively related to willingness to participate in biobanking through religious acceptance (β = 0.20, *p* < 0.01) and perceived benefits (β = 0.32, *p* < 0.001) (Fig. 2). This indicates that when respondents are more engaged in and “informed” of modern biotechnology, they perceive more benefits from biobanks and agree that their application is acceptable as part of their religion and customs.

It is interesting to note that the SEM results show that the respondents who are more attached to their religions tended to be more critical of biobanks. Those who claim to be highly religious tended to perceive more benefits of biobanking (β = 0.11, *p* < 0.05) while at the same time foreseeing certain risk issues underlying its application (β = 0.14, *p* < 0.05) (Fig. 2). Trust in key players is strongly positively associated with perceived benefits (β = 0.34, *p* < 0.001), indicating that respondents with more confidence in key players, such as producers (including those who operate biobanks), scientists and policy makers, tend to view biobanks as beneficial (Fig. 2). Moreover, attitudes towards technology are positively associated with issues of data and specimen protection (β = 0.32, *p* < 0.001), suggesting that those who hold a negative predisposition towards technology are more likely to perceive greater risks related to biobanking (Fig. 2).

## Discussion

It is interesting to see that Malaysian stakeholders in the Klang Valley tended to recognize moderate benefits of biobanks while at the same time being cautious and perceiving issues of data and specimen protection in biobanks as moderately important. Previous studies have shown that respondents who exhibit concern regarding data privacy and protection also recognize certain benefits of biobanks [3, 17]. Niu [70] also suggested that some negative consequences of biobanks are too minor to be taken into consideration, especially when compared to the potential benefits of their application. Despite moderate concerns relating to biobank issues, the mean score for the variable was found to be much lower than the perceived benefits, thus translating into a moderate willingness to participate in biobanking overall. The findings of this study is not surprising as past studies have cited that the public worldwide were critical of biotechnology-related applications and products [37, 43, 54, 71, 72].

This finding is supported by the earlier studies on biotechnology application in Malaysia where perceived benefits have been identified as the strongest predictor of attitudes towards GM soybeans [37] and GM mosquitos [73] and as one of the direct predictors of attitudes towards GM rice [74]. Further, Gaskell et al. [38] also suggest that perceived benefits act as a pre-requisite determinant of support for various biotechnology applications, while Lemke et al. [41] show that Americans’ willingness to participate in biobanks is driven by an interest in bettering society by curing disease. A positive relationship between the perceived benefits of research and support for biobanks has also been found in studies of the United States [2] and Australia [25].

The negative relationship between issues of data and specimen protection and willingness to participate suggests that Malaysian stakeholders in the Klang Valley tend to support biobank application when they take less issue with the issues mentioned above. Gaskell et al. [16] previously found that Europeans’ readiness to accept broad consent and willingness to participate in biobank research are dependent on a range of interconnected factors including views concerning privacy and data security issues and issues of benefit sharing. Concerns regarding genetic discrimination resulting from the sharing of genetic information were also mentioned by participants as a barrier to participation [41]. However, it is interesting to note that the effect of privacy concerns on willingness to participate in biobanking could also be mitigated by other factors, such as the introduction of incentives for participants. A study conducted in the USA suggests that concerns about privacy were related to a lower willingness to participate only when respondents were briefed that they would receive token for participating and would not be informed about the research results individually. Among respondents who were told that they would receive $200 or individual research results, privacy concerns were not related to levels of willingness [26]. Public concern about privacy issues also does not necessarily cause rejection of biobanks [2, 16, 26], as focus group participants in both studies expected biobanks to offer the best possible protections if data were abused by insurance companies or employers. Furthermore, people’s concern regarding data security and privacy protection may be attenuated by trust and beliefs concerning benefit sharing [16].

Trust in key players involved in the development or regulation of modern biotechnology was also found to have direct influence on willingness to participate in biobanking. This finding is consistent with some earlier studies. For example, a hypothetical Swedish study of the general population found that high levels of public trust in science, university researchers and hospitals has been key to successful biobank research as well as dictating public willingness to provide blood samples [24]. In an Australian study, trust in biobanks was found to be one of the determinant of intentions to participate in biobank research [25]. The effect of trust was reported to be almost ten times more important than beliefs regarding benefits and five times more important than levels of comfort with blood donation and deoxyribonucleic acid (DNA) analysis [25]. Gaskell et al. also found that trust in the socio-political system and in key actors and institutions involved in biobanks also determine public willingness to participate in biobank research [16]. Trust in key players is strongly positively associated with perceived benefits, indicating that when respondents have more confidence in key players, such as producers (including those who operate the biobanks), scientists and policy makers, they were more inclined to rate biobanks as beneficial. This finding corresponds with those of Amin et al. [37] and Amin and Hashim [73] who also reported a relationship between confidence in key actors and perceived benefits. Previously, trust in institutions using gene technology has been shown to have a positive impact on perceived benefits and a negative influence on perceived risks of technology [39]. Gaskell et al. [38], Ghasemi et al. [74] and Hossain and Onyango [75] reported that trust in key players such as the government and scientists is shown to be an important determinant of acceptance of GM technologies. Critchley et al. [25], however, found trust in biobanking to be the most important determinant of intentions to participate in biobanks with an estimate almost ten times that found for benefit beliefs and five times that found for comfort with blood donation and DNA analysis.

The results of this study indicates that Malaysian stakeholders in the Klang Valley also consider their religious judgements together with perceived benefits, issues of data and specimen protection, and trust in key players, when deciding whether or not to support biobank application. This finding is congruent with a study by Amin et al. [76] that reports a positive and direct association between religious acceptance and attitudes towards modern biotechnology application. Although perceived benefits are the most important predictor of overall support for biobanks, Malaysian stakeholders also consider their religious views when determining whether to support biobank application. In a conservative country such as Malaysia, this is not surprising since religion and local customs serve as an important aspect in people’s daily lives. In fact, as Malaysian stakeholders describe themselves as highly religious (Table 2), the significant role of religious views is clearly observed when stakeholders are deciding whether to support biobanks. Igbe and Adebamowo [17] once reported that focus group participants in Nigeria experience no resistance from their religion and culture regarding biobank research while Nasrella and Clark [18] also found that Qatari nationals view volunteering for biobanks as a charitable act compatible with Islam that helps future generations. Ahram et al. [51] also reported that Jordanians viewed that religious permission to make biospecimen donations for research purposes has a positive influence on biobank participation. It is interesting to note that SEM results show that respondents who are more attached to their religions tend to be more critical of biobanks. Those who describe themselves as highly religious tend to perceive more benefits of biobanks while at the same time also foreseeing some risk related to their application. The same result has been derived by previous works on biotechnology application in Malaysia [37]. Sanderson et al. [20] also reported on the association between low levels of religiosity and public willingness to participate in the United States.

In addition to direct relationships, stakeholders’ willingness to engage in biobanking also involves the interplay of other factors. In this study, the issue of data and specimen protection was found to be negatively associated with religious acceptance, indicating that stakeholders who view biobanks as risky tend to view their application as less acceptable according to their religious and cultural views. Engagement is also indirectly and positively related to the willingness to participate in biobanking through religious acceptance and perceived benefits. This result indicates that when respondents are more engaged and “informed” with modern biotechnology, they perceive more benefits from biobanks and agree that their application is acceptable according to their religion and customs. This finding highlights the importance of knowledge, awareness and information seeking behaviour in developing positive attitudes towards biobanks, as stakeholders’ only form attitudes about technologies after they have amassed relevant information [34]. It is reported that perceived knowledge of genetic modification technology plays a significant role in the acceptance of GM products [27]. Ahram et al. [14] found that publics with positive perceptions of the level of scientific research conducted in Jordan express a positive attitude towards research investment and are likely to participate in biobanking, while a Swedish study by Lemke et al. [41] found that a lack of information and understanding regarding genetic research may create a barrier to participation in biobanking.

Moreover, attitudes towards technology are positively related with the issue of data and specimen protection, suggesting that those with a negative predisposition towards technology are more likely to perceive higher risks related to biobanking. These relationships are backed by past studies showing that respondents with negative predispositions towards science and technology have higher concerns about biotechnology and view biotechnology applications as unfamiliar and risky [73]. In addition, the UK public is sceptical of the capacities of even sophisticated technologies for protecting data and are also concerned about the personal data security of biobanks, believing that what is coded with a computer can always be decoded with a computer [8].

It is pertinent to admit the limitations of the proposed model in demonstrating stakeholders’ willingness to participate in biobanking. First, it is not recommended to generalize the model beyond the studied population, as the specific populations of the stakeholders involved mostly remained unknown. The model was generated using a combined sample of various stakeholder representatives, such as policy makers, scientists, representatives of NGOs, religious experts and consumers, due to our limited capacity to find more respondents to represent each category. Despite such limitations, the model proposed in this study was validated using the chosen measured variables. Several steps have been given due consideration in justifying the choice of indicators, which represent specific latent variables, and these indicators were recommended by previous related research. Furthermore, standard testing for the validity and reliability of the measures was carried out, and only good indicators were selected. It has to be acknowledged that influences of the factors may vary over time; therefore, further research should be carried out over a different time frame to judge the effects of time. At the same time, the model is valuable to provide an initial account of important predictors of the Malaysian public’s intentions to participate in biobanking, as the respondents mirror the diverse backgrounds of the Malaysian population. Over the last six years since the data were collected there have been no dramatic changes in the development of biobanks in Malaysia. The country’s current focus on biobanks remains essentially the same. However, this model should be verified using samples from other regions of Malaysia and with more specialized stakeholder groups to confirm whether the results are comparable and can be generalized.

## Conclusions

The findings of this study confirm that stakeholders’ willingness to participate in biobanking to improve the prevention, diagnosis and treatment of diseases is a complex matter that should be viewed as part of a multidimensional process. These predictors are useful in understanding social acceptance of biobanks. Public willingness to participate in biobanking is warranted when four direct predictors are seriously considered: the specific application-linked perceptions of biobank benefits, issues of data and specimen protection, religious acceptance, and trust in key players. Notably, the associated benefits, as a precondition of support for biobanks, must be clearly presented to participants. Issues of data and specimen protection must be sufficiently addressed to increase public willingness to participate in biobanking. This calls for appropriate governance by the relevant authorities to ensure that the misuse of data will not happen. Competent governance will portray key players as doing good for society, which in turn will increase public trust. Once trust is achieved, this will enhance public perceptions of benefits and public willingness to participate. At the same time, there must be a clear account of religious perspectives of biobanking, as religious endorsement will increase public willingness to participate. As can be observed from the study findings, religion is an important aspect of life in Malaysia, and people who are more religious tend to be more critical of biobanks. Therefore, it is paramount to have relevant religious authorities specify religious rulings on biobanks. The indirect yet crucial roles of general factors such as religiosity, general attitudes towards technology and engagement are affirmed in this study. Before individuals can appreciate specific technological applications such as biobanks, they must be able to appreciate technology in general. The latest developments in science, technology and healthcare must be covered more in mainstream newspapers and on radio and television shows, and more awareness programmes such as public forums and exhibitions must be carried out. These efforts will help boost public engagement with the latest technology, and at the same time, more direct interaction between key players and public programmes will enhance public trust.

## Additional file

Additional file 1: **Appendix** Survey instrument. (DOCX 47 kb)

## Abbreviations

AGFI: Adjusted goodness of fit index
AVE: Average variance extracted
CFA: Confirmatory factor analysis
CFI: Comparative fit index
DNA: Deoxyribonucleic acid
GFI: Goodness of fit index
GLM: General Linear Modelling
GM: Genetically modified
IBM SPSS Amos: International Business Machines Corporation Software Package for Social Sciences, Analysis of a Moment Structures
MI: Modification indices
NFI: Normed fit index
NGOs: Non-governmental organizations
NNFI: Non-normed fit index
RMSEA: Root mean square error of approximation
SEM: Structural equation modelling
UK: United Kingdom
USA: United States of America
**α**: Alpha

## References

[1] Jamal R, Syed Zakaria SZ, Kamaruddin MA, Abd Jalal N, Ismail N, Mohd Kamil N, Abdullah N, Baharudin N, Hussin NH, Othman H, Mahadi NM (2015). Cohort profile: the Malaysian cohort (TMC) project: a prospective study of non-communicable diseases in a multi-ethnic population. Int J Epidemiol.

[2] Gaskell G, Stares S, Allansdottir A, Allum N, Castro P, Esmer Y, Fischler C, Jackson J, Kronberger N, Hampel J, Mejlgaard N, Quintanilha A, Rammer A, Revuelta G, Stoneman P, Torgersen H, Wagner W (2010). Europeans and biotechnology in 2010 - winds of change?. A report to the European Commission’s directorate-general for research. European Commission.

[3] Kaul P, Jaiswal P (2018). Biobanking market - global opportunity analysis and industry forecast, 2018–2025.

[4] The Malaysian Cohort. Objective. 2014a. http://www.ukm.my/mycohort/ms/objektif/. Accessed 20 Feb 2014.

[5] Bin Abdul Aziz MF. Malaysian biobanks: is the current governance framework adequate?. The Centre for law and Ethics in science and technology (CELEST)newsletter. 2018a. Issue 1: 2–6. http://law.um.edu.my/celest-newsletter. Accessed 18 Oct 2018.

[6] Kaufman D, Geller G, Leroy L, Murphy J, Scott J, Hudson K (2008). Ethical implications of including children in a large biobank for genetic-epidemiologic research: a qualitative study of public opinion. Am J Med Genet C: Semin Med Genet.

[7] National Human Genome Research Institute. Design Considerations for a Potential United States Population-Based Cohort to Determine the Relationships among Genes, Environment, and Health: Recommendations of an Expert Panel. 2004. https://www.genome.gov/pages/about/od/reportspublications/potentialuscohort.pdf. Accessed 8 Feb 2014.

[8] Hobbs A, Starkbaum J, Gottweis U, Wichmann HE, Gottweis H (2012). The privacy-reciprocity connection in biobanking: comparing German with UK strategies. Public Health Genomics..

[9] Beyleveld D, Buchanan JA (2007). Consent in the law.

[10] Austin MA, Harding S, McElroy C (2003). Genebanks: a comparison of eight proposed international genetic databases. Public Health Genomics..

[11] Mancini J, Pellegrini I, Viret F, Vey N, Daufresne LM, Chabannon C, Julian-Reynier C (2011). Consent for biobanking: assessing the understanding and views of cancer patients. J Natl Cancer Inst.

[12] Giesbertz NA, Bredenoord AL, Van Delden JJ (2012). Inclusion of residual tissue in biobanks: opt-in or opt-out?. PLoS Biol.

[13] World Health Organization (2009). Proposed international guidelines on ethical issues in medical genetics and genetic services.

[14] Ahram M, Othman A, Shahrouri M (2013). Public support and consent preference for biomedical research and biobanking in Jordan. Eur J Hum Genet.

[15] Al-Jumah MA, Abolfotouh MA (2011). Public perception and attitude of saudis toward organ and tissue donation. Biopreservation and biobanking.

[16] Gaskell G, Gottweis H, Starkbaum J, Broerse JE, Gerber M, Gottweis U, Hobbs A, Ilpo H, Pashou M, Snell K, Soulier A (2011). Publics and Biobanks in Europe: Explaining Heterogeneity. LSG Working Papers 2011/2–October 5.

[17] Igbe MA, Adebamowo CA (2012). Qualitative study of knowledge and attitudes to biobanking among lay persons in Nigeria. BMC medical ethics.

[18] Nasrella E, Clark B. Public attitudes towards participation in biobank Qatar. InQatar Foundation Annual Research Forum. 2012;(2012):BMP78.

[19] Nicol D, Critchley C, McWhirter R, Whitton T (2016). Understanding public reactions to commercialization of biobanks and use of biobank resources. Soc Sci Med.

[20] Sanderson SC, Brothers KB, Mercaldo ND, Clayton EW, Antommaria AH, Aufox SA, Brilliant MH, Campos D, Carrell DS, Connolly J, Conway P (2017). Public attitudes toward consent and data sharing in biobank research: a large multi-site experimental survey in the US. Am J Hum Genet.

[21] Van Draanen J, Davidson P, Bour-Jordan H, Bowman-Carpio L, Boyle D, Dubinett S, Gardner B, Gardner J, McFall C, Mercola D, Nakazono T (2017). Assessing researcher needs for a virtual biobank. Biopreservation and Biobanking.

[22] Murad AM, Myers MF, Thompson SD, Fisher R, Antommaria AH (2017). A qualitative study of adolescents’ understanding of biobanks and their attitudes toward participation, re-contact, and data sharing. Am J Med Genet A.

[23] Critchley C, Nicol D, McWhirter R (2017). Identifying public expectations of genetic biobanks. Public Underst Sci.

[24] Kettis-Lindblad Å, Ring L, Viberth E, Hansson MG (2005). Genetic research and donation of tissue samples to biobanks. What do potential sample donors in the Swedish general public think?. The European Journal of Public Health.

[25] Critchley CR, Nicol D, Otlowski MF, Stranger MJ (2010). Predicting intention to biobank: a national survey. The European Journal of Public Health..

[26] Kaufman DJ, Murphy-Bollinger J, Scott J, Hudson KL (2009). Public opinion about the importance of privacy in biobank research. Am J Hum Genet.

[27] Bredahl L (1999). Consumerss’ cognitions with regard to genetically modified foods. Results of a qualitative study in four countries. Appetite.

[28] Bin Abdul Aziz MF, Morrison M, Kaye J. Regulating human stem cell research and therapy in low-and middle-income countries: Malaysian perspectives. New Genetics and Society. 2018a Jan;37(1):2, 2–0.

[29] Hashim H, Amin L, Mahadi Z, Ismail K (2017). Stakeholders’ attitudes towards biobanks in Malaysia. Akademika.

[30] Pardo R, Midden C, Miller JD (2002). Attitudes toward biotechnology in the European Union. J Biotechnol.

[31] Fishbein M, Ajzen I (1975). Belief, attitude, intention and behavior: an introduction to theory and research.

[32] Chen MF, Li HL (2007). The consumer’s attitude toward genetically modified foods in Taiwan. Food Qual Prefer.

[33] Grunert KG, Lähteenmäki L, Nielsen NA, Poulsen JB, Ueland O, Åström A (2001). Consumer perceptions of food products involving genetic modification—results from a qualitative study in four Nordic countries. Food Qual Prefer.

[34] Kelley J (1995). Public perceptions of genetic engineering: Australia, 1994.

[35] Nicholas B. The ethical issues of genetic modification. Reflections on the Use of Human. 2000.

[36] Amin L, Azlan A, Gausman M, Ahmad J, Samian A, Haron M, Sidik N (2010). Ethical perception of modern biotechnology with special focus on genetically modified food among Muslims in Malaysia. Asia Pac J Mol Biol Biotechnol.

[37] Amin L, Md AJ, Md JJ, Nor AR, Osman M, Mahadi NM (2011). Factors influencing Malaysian public attitudes to agro-biotechnology. Public Underst Sci.

[38] Gaskell G, Allum N, Stares S, Fjæstad B, Öhman S, Olofsson A. Europeans and biotechnology in 2002-Eurobarometer 58.0: A report to the EC Directorate General for Research from the project" Life Sci in European Society". 2013.

[39] Siegrist M (2000). The influence of trust and perceptions of risks and benefits on the acceptance of gene technology. Risk Anal.

[40] Cheung MW, Chan W (2005). Meta-analytic structural equation modeling: a two-stage approach. Psychol Methods.

[41] Lemke AA, Wolf WA, Hebert-Beirne J, Smith ME (2010). Public and biobank participant attitudes toward genetic research participation and data sharing. Public Health Genomics..

[42] Christoph IB, Bruhn M, Roosen J (2008). Knowledge, attitudes towards and acceptability of genetic modification in Germany. Appetite.

[43] Amin L, Jahi JM, Nor AR, Osman M, Mahadi NM (2006). Uncovering factors influencing Malaysian public attitude towards modern biotechnology. Asia Pacific Journal of Molecular Biology & Biotechnology..

[44] Frewer LJ, Howard C, Aaron JI (1998). Consumer acceptance of transgenic crops. Pestic Sci.

[45] Einsiedel EF (1997). Biotechnology and the Canadian public: report on a 1997 National Survey and some international comparisons. University of Calgary, Alberta. Gaskell et al. 2000. Biotechnology and the European public. Nat Biotechnol.

[46] Rowe G (2004). How can genetically modified foods be made publicly acceptable?. Trends Biotechnol.

[47] Sparks P, Shepherd R (1994). Public perceptions of the potential hazards associated with food production and food consumption: an empirical study. Risk Anal.

[48] Hansen J, Holm L, Frewer L, Robinson P, Sandøe P (2003). Beyond the knowledge deficit: recent research into lay and expert attitudes to food risks. Appetite.

[49] Saifuddeen SM, Rahman NN, Isa NM, Baharuddin A (2014). Maqasid al-Shariah as a complementary framework to conventional bioethics. Sci Eng Ethics.

[50] Ahram M, Othman A, Shahrouri M, Mustafa E (2014). Factors influencing public participation in biobanking. Eur J Hum Genet.

[51] Medical Research Council and Wellcome Trust. Public Perceptions of the Collection of Human Biological Samples. London: Medical Research Council; 2001. http://www.ukbiobank.ac.uk/wp-content/uploads/2011/07/Public-Perceptions-Collection-Human-Biological-Samples.pdf. Accessed 1 Feb 2014

[52] Wong ML, Chia KS, Wee S, Chia SE, Lee J, Koh WP, Shen HM, Thumboo J, Sofjan D (2004). Concerns over participation in genetic research among Malay-Muslims, Chinese and Indians in Singapore: a focus group study. Public Health Genomics.

[53] Rohrmann B (1994). Risk perception of different societal groups: Australian findings and cross-national comparisons. Aust J Psychol.

[54] Macer D (2001). Bioethics: perceptions of biotechnology and policy implications. Int J Biotechnol.

[55] Gaskell G, Allum N, Bauer M, Durant J, Allansdottir A, Bonfadelli H, Boy D, De Cheveigné S, Fjaestad B, Gutteling JM, Hampel J (2000). Biotechnology and the European public. Nat Biotechnol.

[56] Golob TF (2003). Structural equation modeling for travel behavior research. Transp Res B Methodol.

[57] Black WC HJF, Babin BJ, Anderson RE (2010). multivariate data analysis, a global perspective.

[58] Anderson JC, Gerbing DW (1988). Structural equation modeling in practice: a review and recommended two-step approach. Psychol Bull.

[59] Batista JM, Coenders G. Modelos de ecuaciones estructurales. Int J Psychol. 45(2):131–9.

[60] Lévy JP, Varela J (2003). Análisis multivariable para las ciencias sociales.

[61] Luque Martínez T, Barrio García SD (2000). Técnicas de análisis de datos en investigación de mercados.

[62] Lei PW, Wu Q (2007). Introduction to structural equation modeling: issues and practical considerations. Educ Meas Issues Pract.

[63] Jöreskog KG, Sörbom DL (1996). LISREL 8: User's reference guide.

[64] Byrne BM. Structural equation modeling with AMOS: basic concepts, applications, and programming: Routledge; 2016.

[65] Hair JF, Black WC, Babin BJ, Anderson RE, Tatham RL (2006). Multivariate data analysis (Vol. 6).

[66] Arbuckle JL, Users’Guide WW (1999). Version 4.0.

[67] Lomax RG. Schumacker RE: A beginner's guide to structural equation modeling. psychology press; 2004.

[68] Costa-Font M, Gil JM (2009). Structural equation modelling of consumer acceptance of genetically modified (GM) food in the Mediterranean Europe: a cross country study. Food Qual Prefer.

[69] Carmines E, McIver J. Analyzing models with unobserved variables: Analysis of covariance structures. In: Bohrnstedt GW, Borgatta FF, editors. Social measurement-current issues: Sage Publications Inc; 1981.

[70] Niu H. Benefits, risks and Trust in Human Biobanks. 2009. file:///C:/Users/LATIIFAH/Downloads/Huei%20Chih%20Niu%20(1).pdf. Accessed 2 Jan 2015.

[71] Chern WS, Rickertsen K, Tsuboi N, Fu TT. Consumer acceptance and willingness to pay for genetically modified vegetable oil and salmon: A multiple-country assessment. AgBioForum. 2002;5(3):105–112.

[72] Haro MN. Sustainability aspects of applying GMOs in aquaculture (Master's thesis, Norwegian University of Life Sciences, Ås). 2012

[73] Amin L, Hashim H (2015). Factors influencing stakeholders attitudes toward genetically modified aedes mosquito. Sci Eng Ethics.

[74] Ghasemi S, Karami E, Azadi H (2013). Knowledge, attitudes and behavioral intentions of agricultural professionals toward genetically modified (GM) foods: a case study in Southwest Iran. Sci Eng Ethics.

[75] Hossain F, Onyango B (2004). Product attributes and consumer acceptance of nutritionally enhanced genetically modified foods. Int J Consum Stud.

[76] Amin L, Azad MA, Ahmad Azlan NA, Zulkifli F (2014). Factors influencing stakeholders' attitudes toward cross-kingdom gene transfer in rice. New Genetics and Society.

