# Supplementary material for: Determinants of the willingness to participate in biobanking among Malaysian stakeholders in the Klang Valley
Source: BMC Med Res Methodol. 2018 Dec 5;18:163. doi: 10.1186/s12874-018-0619-2 (PMC6282379; doi:10.1186/s12874-018-0619-2)
Supplement: Supplementary file 1 — Appendix Survey instrument. (DOCX 47 kb) [file 12874_2018_619_MOESM1_ESM.docx]

**SURVEY INSTRUMENT**

**Engagement**

1. **Past and intended behaviour**

To what extent, do you agree with the following statements about modern biotechnology?

|  | Strongly Strongly  disagree agree |
| --- | --- |
| 1. Before this survey, I often talked about biotechnology with other people. | 1 2 3 4 5 6 7 |
| 1. I often involved in public discussions or hearings about modern biotechnology. | 1 2 3 4 5 6 7 |
| 1. I often take the time to read articles from newspapers or magazines, or watch television program on modern biotechnology. | 1 2 3 4 5 6 7 |
| 1. I often read about modern biotechnology from the internet. | 1 2 3 4 5 6 7 |
| 1. I often participate in online discussions about modern biotechnology through online chatting, forum, social networks, blog, etc. | 1 2 3 4 5 6 7 |

1. **Awareness**

Have you heard about the following things before this survey?

|  | Yes | No |
| --- | --- | --- |
| 1. Cloned animal such as the sheep 'Dolly' | 1 | 2 |
| 1. Human genome project to map human genes | 1 | 2 |
| 1. ‘Golden Rice’ which is high in vitamin A content | 1 | 2 |
| 1. Biodiesel as an alternative fuel for vehicles | 1 | 2 |
| 1. Genetically modified mosquitoes to control dengue | 1 | 2 |
| 1. Synthetic biology which creates novel organism that carries out completely new functions | 1 | 2 |
| 1. Biobank which collects and maintains blood, tissues, DNA and personal data from large number of people to improve prevention, diagnosis and treatment of diseases | 1 | 2 |
| 1. Human stem cell to replace diseased cell/tissues | 1 | 2 |
| 1. National Biotechnology Policy in Malaysia | 1 | 2 |
| 1. Bio-safety Act in Malaysia | 1 | 2 |

1. **Knowledge**

For each of the following statements, please indicate whether you think it is true or false.

|  | True | False | Don’t know |
| --- | --- | --- | --- |
| 1. Yeast that is used for making bread is a living organism | 1 | 2 | 3 |
| 1. There are useful bacteria living in our body | 1 | 2 | 3 |
| 1. It is the mother's genes that determine whether a child is a girl | 1 | 2 | 3 |
| 1. Ordinary tomatoes do not contain genes, while genetically modified tomatoes do | 1 | 2 | 3 |
| 1. By eating a genetically modified fruit, a person's genes could become modified | 1 | 2 | 3 |
| 1. It is possible to find out whether a child will have Down's Syndrome during the earlier pregnancy stage | 1 | 2 | 3 |
| 1. Genetically modified plants are always bigger and have more fruits | 1 | 2 | 3 |
| 1. More than half of the human genes are identical to those of chimpanzees | 1 | 2 | 3 |
| 1. The cloning of living things produces genetically identical copies | 1 | 2 | 3 |
| 1. All products resulted from biotechnology processes contain modified genes | 1 | 2 | 3 |

**Trust in key player (items 4-6)**

To what extent that the following institutions/group **have done a good job** for the society?

| Institution/group | Strongly Strongly  disagree agree |
| --- | --- |
| 4. Scientists/researchers from universities and research institutes | 1 2 3 4 5 6 7 |
| 5. Industry/manufacturer of food, pharmaceutical, agriculture | 1 2 3 4 5 6 7 |
| 6. Govt. dept. involved in modern biotechnology regulation such as Dept. of Biosafety, Ministry of Natural Sources and Environment | 1 2 3 4 5 6 7 |

**Attitude to technology (items 7-10)**

Please circle your level of agreement with the following statements

|  | Items | Strongly Strongly  disagree agree | |  |
| --- | --- | --- | --- | --- |
| 7 | Incessant science and technological progress will finally lead to humanity's extermination | 1 2 3 4 5 6 7 | |  |
| 8 | Industry and technology have such an impact on urban life that you can't feel well and happy anymore | 1 2 3 4 5 6 7 | |  |
| 9 | It is detrimental for humanity that the modern world is more and more dependent on technology | 1 2 3 4 5 6 7 | |  |
| 10 | 10. Modern technology has upset the balance of nature | | | 1 2 3 4 5 6 7 |

**Religiosity (items 11-14)**

Please circle your level of agreement with the following statements:

| Items | Strongly Strongly disagree agree |
| --- | --- |
| 11. Religion is important in my life | 1 2 3 4 5 6 7 |
| 12**.** Religious views are important when I have to make decisions about controversial issues | 1 2 3 4 5 6 7 |
| 13. Praying is important in my life | 1 2 3 4 5 6 7 |
| 14. Reading scriptures is important in my life | 1 2 3 4 5 6 7 |

**Religious acceptance (item 15-16), perceived benefit (item 17-20), issue of data and specimen protection (item 22-25),**

**willingness to participate in biobanking (item 26-28)**

Please circle the number that corresponds to your answer for each question.

| Item 15. The following application can be accepted by my religion.  Application: Collecting and maintaining blood, tissues, DNA and personal data from civilians to improve prevention, diagnosis and treatment of diseases. | Not Very  acceptable acceptable  at all  1 2 3 4 5 6 7 | | | | | |  |
| --- | --- | --- | --- | --- | --- | --- | --- |
| Item 16. The following applications can be accepted by my customs.  Application: Collecting and maintaining blood, tissues, DNA and personal data from civilians to improve prevention, diagnosis and treatment of diseases can be accepted by my customs. | Not Very  acceptable acceptable  at all  1 2 3 4 5 6 7 | | | | | | |
| Item 17. The following application will enhance the quality of life of the Malaysian society.  Application: Biobank which collects and maintains blood, tissues, DNA and personal data from civilians to improve prevention, diagnosis and treatment of diseases. | | Strongly Strongly  disagree agree  1 2 3 4 5 6 7 | | |  |  |  |
| Item 18. To what extent does the following products/applications useful to the Malaysian society?  Application: Biobank which collects and maintains blood, tissues, DNA and personal data from civilians to improve prevention, diagnosis and treatment of diseases. | | Not very Very useful useful  1 2 3 4 5 6 7 | | |  |  |  |
| Item 19. The following applications enable the society to solve problems that currently cannot be solved by the traditional method  Application: Collecting and maintaining blood, tissues, DNA and personal data from civilians to improve prevention, diagnosis and treatment of diseases. | | Strongly Strongly disagree agree  1 2 3 4 5 6 7 | | |  |  |  |
| Item 20. The benefits of the following applications exceed their risks.  Application: Collecting and maintaining blood, tissues, DNA and personal data from civilians to improve prevention, diagnosis and treatment of diseases. | | Strongly Strongly disagree agree  1 2 3 4 5 6 7 | | |  |  |  |
| Item 22. Using the following products/applications may give rise to unknown consequences  Application: Biobank which collects and maintains blood, tissues, DNA and personal data from civilians to improve prevention, diagnosis and treatment of diseases. | | Strongly Strongly disagree agree  1 2 3 4 5 6 7 | | |  |  |  |
| Item 24. Are you worried about the ownership issue of biobank data and specimen? | | | Not Very  worried worried  at all  1 2 3 4 5 6 7 | | |  |  |
| Item 25. The probability of misuse of data and specimen by researchers are high | | | Strongly Strongly disagree agree  1 2 3 4 5 6 7 | | |  |  |
| Item 26. I am willing to use/support the following application.  Application: Biobank which collects and maintains blood, tissues, DNA and personal data from civilians to improve prevention, diagnosis and treatment of diseases. | | | Strongly Strongly disagree agree  1 2 3 4 5 6 7 | | |  |  |
| Item 27. I am willing to provide information about myself to biobank | | | Not willing Very at all willing  1 2 3 4 5 6 7 |  |  |  |  |
| Item 28. I am willing to give my blood or tissue sample to biobank | | | Not willing Very at all willing  1 2 3 4 5 6 7 |  |  |  |  |
